# Interactions of Freshwater Cyanobacteria with Bacterial Antagonists

**DOI:** 10.1128/AEM.02634-16

**Published:** 2017-03-17

**Authors:** Omneya Ahmed Osman, Sara Beier, Manfred Grabherr, Stefan Bertilsson

**Affiliations:** a Department of Ecology and Genetics, Limnology, and Science for Life Laboratory, Uppsala University, Uppsala, Sweden; b Leibniz Institute for Baltic Sea Research, Warnemünde, Germany; c Department of Medical Microbiology and Biochemistry, Bioinformatics Infrastructure for Life Sciences, and Science for Life Laboratory, Uppsala University, Uppsala, Sweden; The University of Tokyo

**Keywords:** coculture interaction, cyanobacteria, metatranscriptome

## Abstract

Cyanobacterial and algal mass development, or blooms, have severe effects on freshwater and marine systems around the world. Many of these phototrophs produce a variety of potent toxins, contribute to oxygen depletion, and affect water quality in several ways. Coexisting antagonists, such as cyanolytic bacteria, hold the potential to suppress, or even terminate, such blooms, yet the nature of this interaction is not well studied. We isolated 31 cyanolytic bacteria affiliated with the genera Pseudomonas, Stenotrophomonas, Acinetobacter, and Delftia from three eutrophic freshwater lakes in Sweden and selected four phylogenetically diverse bacterial strains with strong-to-moderate lytic activity. To characterize their functional responses to the presence of cyanobacteria, we performed RNA sequencing (RNA-Seq) experiments on coculture incubations, with an initial predator-prey ratio of 1:1. Genes involved in central cellular pathways, stress-related heat or cold shock proteins, and antitoxin genes were highly expressed in both heterotrophs and cyanobacteria. Heterotrophs in coculture expressed genes involved in cell motility, signal transduction, and putative lytic activity. l,d-Transpeptidase was the only significantly upregulated lytic gene in Stenotrophomonas rhizophila EK20. Heterotrophs also shifted their central metabolism from the tricarboxylic acid cycle to the glyoxylate shunt. Concurrently, cyanobacteria clearly show contrasting antagonistic interactions with the four tested heterotrophic strains, which is also reflected in the physical attachment to their cells. In conclusion, antagonistic interactions with cyanobacteria were initiated within 24 h, and expression profiles suggest varied responses for the different cyanobacteria and studied cyanolytes.

**IMPORTANCE** Here, we present how gene expression profiles can be used to reveal interactions between bloom-forming freshwater cyanobacteria and antagonistic heterotrophic bacteria. Species-specific responses in both heterotrophs and cyanobacteria were identified. The study contributes to a better understanding of the interspecies cellular interactions underpinning the persistence and collapse of cyanobacterial blooms.

## INTRODUCTION

Many nutrient-rich freshwater ecosystems experience episodic mass development of cyanobacteria. These blooms influence water quality through the shear quantity of organisms and the release of toxic compounds (1–3). Trophic interactions can lead to cyanobacterial toxins being accumulated in aquatic organisms (e.g., mussels, crayfish, and fish), which are subsequently passed on to humans (4). Microcystis is one of the most widespread and problematic toxin-producing freshwater cyanobacteria. Its toxin, microcystin, can cause liver damage in domestic animals and has a number of severe impacts on lake biota (5). Aphanizomenon flos-aquae is another widespread cyanobacterium, with several strains having the capacity to produce and release toxins that cause fish mortality (6, 7).

The control of algal blooms has previously been facilitated with mechanical methods, such as filtration, ultrasound, and electrolysis, or the addition of toxic chemicals, such as copper sulfate or sodium hypochlorites (8). However, these methods are expensive and not practical for the elimination of algal blooms at the ecosystem scale. The use of biological control agents, such as viruses and predatory bacteria, has been proposed as an alternative strategy to counteract the harmful effects of algal blooms, but the efficacy of this approach is yet to be demonstrated.

Several phylogenetically diverse heterotrophic bacteria within the Proteobacteria, Bacteroides, Firmicutes, and Actinobacteria have displayed antagonistic activities against various cyanobacteria (9–12). These antagonistic bacteria exert lytic activity via mechanisms, including parasitism, antibiosis after host entrapment, or contact lysis (13). For example, close attachment of Streptomyces neyagawaensis to cyanobacterial cells is known to cause efficient lysis after the production of antimicroalgal compounds (10). Other examples include the endoparasitic strain Bdellovibrio bacteriovorous, which was reported to cause lysis of Phormidium luridum by the secretion of extracellular compounds (12, 14) and heterotrophic bacterial production of the antimicroalgal compound β-cyanoalanine (l-CNAla) to control toxin-producing cyanobacteria (15). In the study by Yoshikawa et al. (15), the authors showed that l-CNAla inhibits the growth of some cyanobacterial strains, such as Synechococcus sp. strain CSIRO 94 and Microcystis aeruginosa NIES-298, but not green algae, dinoflagellates, or diatoms.

One powerful approach to map the functional response of microorganisms to environmental cues or interactions is to broadly sequence the combined transcriptome of the full set of interacting organisms. Compared to a metagenomic approach, the major advantage of metatranscriptomic analyses is the possibility to observe actively expressed genes at a certain time point, allowing for the prediction of changes in metabolic pathways and other functional responses (16–18). Recent advances in next-generation RNA sequencing mean that millions of reads can be generated and either be mapped to reference genomes or assembled de novo, making this approach tractable and affordable (19, 20).

We applied a metatranscriptomic approach in the present study, using the input experiment of axenic cultures of Microcystis aeruginosa PCC 7941 and Aphanizomenon flos-aquae PCC 7905, challenged with different confirmed freshwater lake antagonistic or lytic bacteria. The combined mRNA pools, expressed by the heterotrophic antagonistic bacteria cocultured with Microcystis aeruginosa PCC 7941 or Aphanizomenon flos-aquae PCC 7905, were characterized by RNA sequencing. The aim was to uncover the mechanisms underlying the cyanolytic bacterial interactions while at the same time identifying the cyanobacterial response to such pressures.

## RESULTS

### Isolation of cyanolytic bacteria.

Most of the cyanolytic bacteria were from Lake Ekoln and Lake Erken, while only eight strains were from Lake Funbosjön. All of the 31 isolates selected for further characterization propagated well on BG11 medium supplemented with 0.2% Casitone. Twenty of these isolates had high 16S rRNA identity to Pseudomonas species (99 to 100% identity), while 11 isolates showed an equal level of identity to other taxa (Delftia, Stenotrophomonas, Acinetobacter, Marinobacter, and Limnobacter). The 31 isolates displayed high-to-moderate lytic activity with either defined or diffuse lytic zones (see Table S2 in the supplemental material).

### Genomic features of experimental cyanolytic bacteria.

The 16S rRNA gene analysis of the four cyanolytic bacteria selected for further experiments matched with 100% identity to Stenotrophomonas rhizophila (1,527-bp alignment), Pseudomonas putida (1,144 bp), and Acinetobacter beijerinckii (1,518 bp) and 99% identity to Delftia sp. (1,519 bp). The genome statistics and metabolisms of the four strains are summarized in Tables 1 and 2. Inspection of the genomes revealed that the four cyanolytic bacteria are all heterotrophs, with auxotrophic requirement for specific amino acids. S. rhizophila EK20, Delftia sp. strain F45, and A. beijerinckii F107 are auxotrophs for 12 to 17 amino acids (such as l-lysine, l-histidine, and l-tyrosine) and prototrophs for 3 to 4 amino acids (such as glycine and glutamate). Pseudomonas putida EK59 appeared to be auxotrophic for only five amino acids. In each of the four assemblies, about 98% of the genome consists of protein-coding genes, 70 to 80% of which could be functionally annotated as Clusters of Orthologous Groups (COGs) or protein families (Pfams). Most of the basic cellular and metabolic functions were successfully annotated (Tables 1 and 2). For Delftia sp. F45, P. putida EK59, and *S*. rhizophila EK20, >100 genes involved in cell motility and extracellular structures were identified, while few genes coding for cell motility and extracellular structures were identified in the A. beijerinckii F107 genome (20).

**TABLE 1:** Genome statistics of Acinetobacter beijerinckii F107, Delftia sp. strain F45, Pseudomonas putida EK59, and Stenotrophomonas rhizophila EK20

Genome statistics	Acinetobacter beijerinckii F107		Delftia sp. F45		Pseudomonas putida EK59		Stenotrophomonas rhizophila EK20	
	No.	% of total	No.	% of total	No.	% of total	No.	% of total
Total DNA bases	2,304,418	100	5,792,729	100	4,767,657	100	3,665,436	100
DNA-coding bases	2,029,954	88.09	5,216,926	90.06	4,311,136	90.42	3,319,715	90.57
DNA G+C bases	885,232	38.41	3,871,767	66.84	2,962,115	62.13	2,437,651	66.50
DNA scaffolds	594	100.00	391	100.00	530	100.00	384	100
Total genes	2,624	100.00	5,381	100.00	4,730	100.00	3,583	100
Protein-coding genes	2,578	98.25	5,304	98.57	4,614	97.55	3,518	98.19
With function prediction	1,881	71.68	4,337	81.60	3,708	78.39	2,762	77.09
Without function prediction	697	26.56	967	17.97	906	19.15	756	21.10
With enzymes	545	20.77	1,167	21.69	1,079	22.81	820	22.89
Connected to KEGG pathways	530	20.20	1,387	25.78	1,246	26.34	906	25.29
Connected to KEGG Orthology	1,033	39.37	2,436	45.27	2,312	48.88	1,638	45.72
Connected to transporter classification	272	10.37	920	17.10	691	14.61	406	61.76
With COGs	1,349	51.41	3,669	68.18	3,078	65.07	2,213	
With KOGs	409	15.59	914	16.99	796	16.83	618	17.25
With Pfam	1,994	75.99	4,516	83.92	3,946	83.42	2,903	81.02
With TIGRfam	743	28.32	1,472	27.36	1,491	31.52	1,076	30.03
With InterPro	1,271	48.44	3,039	56.48	2,593	54.82	1,883	52.55
Genes in biosynthetic clusters	38	1.45	165	3.07	155	3.28	38	1.06
Fused protein-coding genes	41	1.56	118	2.19	122	2.58	87	2.43
Protein-coding genes coding signal peptides	211	8.04	603	11.21	479	10.13	530	14.79
Protein-coding genes coding transmembrane proteins	581	22.14	1,222	22.71	1,050	22.20	888	24.78

**TABLE 2:** COG categories of protein-coding genes predicted in Acinetobacter beijerinckii F107, Delftia sp. F45, Pseudomonas putida EK59, and Stenotrophomonas rhizophila EK20^*a*^

COG category	Acinetobacter beijerinckii F107		Delftia sp. F45		Pseudomonas putida EK59		Stenotrophomonas rhizophila EK20	
	No.	% of total	No.	% of total	No.	% of total	No.	% of total
Amino acid transport and metabolism	125	8.36	395	9.34	379	10.83	181	7.16
Carbohydrate transport and metabolism	43	2.88	195	4.61	166	4.74	129	5.10
Cell cycle control, cell division, chromosome partitioning	22	1.47	26	0.62	33	0.94	29	1.15
Cell motility	20	1.34	112	2.65	101	2.88	106	4.19
Cell wall/membrane/envelope biogenesis	91	6.09	207	4.90	199	5.68	176	6.96
Chromatin structure and dynamics	1	0.07	1	0.02	2	0.06	1	0.04
Coenzyme transport and metabolism	86	5.75	197	4.66	183	5.23	117	4.63
Defense mechanisms	38	2.54	77	1.82	71	2.03	70	2.77
Energy production and conversion	88	5.89	387	9.16	220	6.28	149	5.90
Extracellular structures	12	0.80	47	1.11	26	0.74	38	1.50
Function unknown	94	6.29	188	4.45	205	5.86	154	6.09
General function prediction only	152	10.17	361	8.54	293	8.37	209	8.27
Inorganic ion transport and metabolism	97	6.49	291	6.88	221	6.31	119	4.71
Intracellular trafficking, secretion, and vesicular transport	20	1.34	69	1.63	58	1.66	54	2.14
Lipid transport and metabolism	102	6.82	252	5.96	153	4.37	111	4.39
Mobilome: prophages, transposons	6	0.40	13	0.31	32	0.91	18	0.71
Nucleotide transport and metabolism	47	3.14	79	1.87	76	2.17	59	2.33
Posttranslational modification, protein turnover, and chaperones	60	4.01	144	3.41	128	3.66	126	4.99
RNA processing and modification	2	0.13	2	0.05	1	0.03	1	0.04
Replication, recombination, and repair	48	3.21	102	2.41	101	2.88	89	3.52
Secondary metabolite biosynthesis, transport, and catabolism	39	2.61	146	3.45	94	2.68	62	2.45
Signal transduction mechanisms	52	3.48	285	6.74	255	7.28	167	6.61
Transcription	119	7.96	452	10.69	304	8.68	179	7.08
Translation, ribosomal structure, and biogenesis	131	8.76	199	4.71	200	5.71	183	7.24
Total COG categories	1,495	100	4,227	100	3,501	100	2,527	100

a % of total represents percentage of protein-coding genes of specific COG category against the total number of COG categories.

The Delftia sp. F45, P. putida EK59, and S. rhizophila EK20 genomes contain 70 to 76 genes predicted to be involved in defense mechanisms, while 39 genes with such functions were present in the A. beijerinckii F107 genome. All four heterotrophic genomes contain peroxiredoxin, and most of them (except for A. beijerinckii F107) encode the osmotically inducible protein OsmC.

### Coculture experiment.

**FIG 1:**
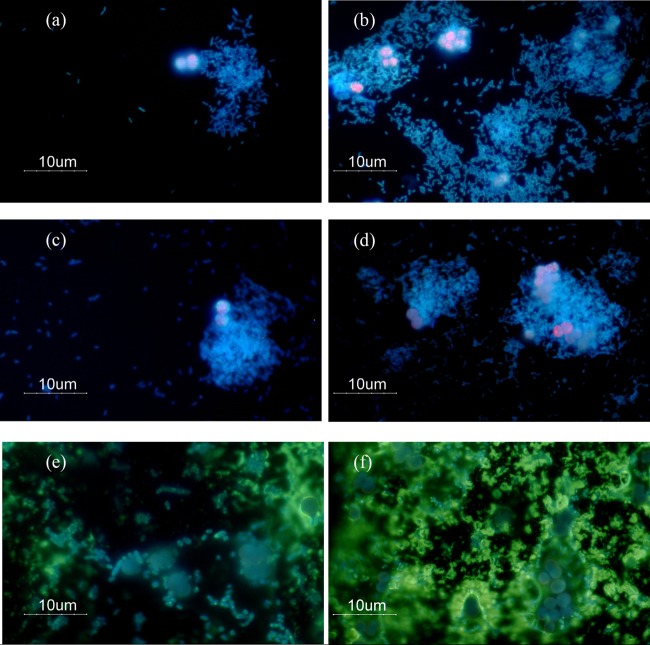
Fluorescence microscopy images of interactions between Stenotrophomonas rhizophila EK20 and Microcystis aeruginosa PCC 7041. (a) Stenotrophomonas cells are in close contact with Microcystis cells after 6 h of incubation time. (b) Stenotrophomonas cells increase in number with complete surrounding of Microcystis cells after 24 h. (c) Stenotrophomonas cells start to form clumps adjacent to Microcystis cells, and cyanobacterial cells enlarge in shape after 72 h. (d) Microcystis-heterotroph aggregates after 96 h. (e and f) Mixed staining with NanoOrange and 4′,6-diamidino-2-phenylindole (DAPI) showed enlarged abnormal shapes of Microcystis cells after 96 h.

Here, cocultures are abbreviated miA, miD, miP, miS, apA, apD, apP, and apS, with the first two letters describing the autotroph genus, followed by the first letter of the heterotroph genus (mi, Microcystis aeruginosa PCC 7941; ap, Aphanizomenon flos-aquae PCC 7905; P, Pseudomonas putida EK59; S, Stenotrophomonas rhizophila EK20; D, Delftia sp. F45; A, Acinetobacter beijerinckii F107). Microscopic observations were first used to describe interactions between the four cyanolytic bacterial strains, and either M. aeruginosa PCC 7941 or *A*. flos-aquae PCC 7905. During the interaction with M. aeruginosa PCC 7941, all four heterotrophic bacteria exhibited very similar visual patterns: first, the heterotrophs adhered to the cyanobacterial cells at 6 h of incubation. Second, the formation of aggregates surrounding M. aeruginosa PCC 7941 was apparent after 24 h of incubation time. Third, many M. aeruginosa PCC 7941 cells featured abnormal and irregular shapes, and extracellular protein structures could be visualized with NanoOrange after 96 h (Fig. 1).

**FIG 2:**
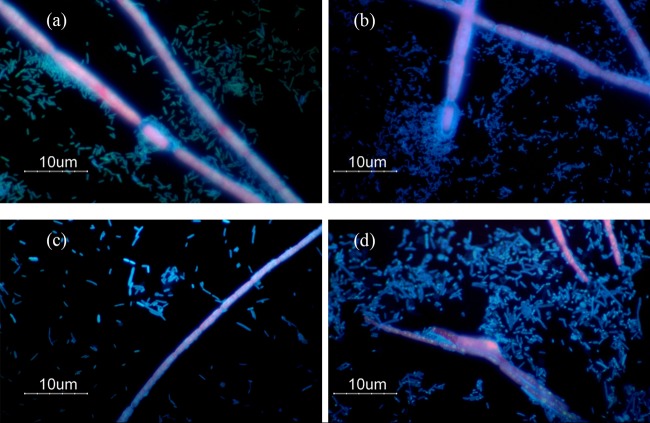
Epifluorescence microscopy images of antagonistic interaction between Stenotrophomonas rhizophila EK20 or Delftia sp. F45 and Aphanizomenon flos-aquae PCC 7905. (a) Stenotrophomonas cell adherence to cyanobacterial heterocysts after 24 h. (b) Stenotrophomonas cell damage of cyanobacterial filaments after 96 h. (c) Delftia cells adhere to Aphanizomenon vegetative cells after 24 h. (d) Delftia cells cause abnormal shape of Aphanizomenon flos-aquae PCC 7905 filaments after 96 h.

During the coculture with the nitrogen-fixing and filamentous cyanobacterium A. flos-aquae PCC 7905, the heterotrophs P. putida EK59 and S. rhizophila EK20 attached to the heterocyst of the filaments after 6 h of incubation. The amount of cells adjacent to the filament had increased after 24 h of contact time, and the disruption of filaments was only starting to become visible after 96 h of incubation. In contrast, Delftia sp. F45 and A. beijerinckii F107 attached mainly to the photosynthetic cells of A. flos-aquae PCC 7905 and increased in numbers between 6 and 24 h of contact time. Abnormal filament shapes were observed after 96 h (Fig. 2).

Despite the apparent strong visible effect of the heterotrophic bacteria on cyanobacterial cell morphology, microscopic cell counts revealed that the growth of the two cyanobacterial strains was not significantly affected (Table S3). The count of heterotrophic bacteria increased during the first 24 h of the experimental incubation and remained stable or slightly increased thereafter. However, as the bacterial population forming aggregates and biofilms adjacent to the cyanobacterial cells could not be accurately counted, these numbers may be underestimates. Irregular shapes of cyanobacterial cells were not counted due to the difficultly in estimating their number in aggregates.

### Highly expressed genes in heterotrophs.

Three COG functional categories, (i) amino acid transport and metabolism, (ii) translation, ribosomal structure, and biogenesis, and (iii) energy production and conversion, were among the most highly expressed genes in all heterotroph cocultures (Table 3). For representative genes of each COG functional category, see Table S4.

**TABLE 3:** Number of highly expressed genes by the four heterotrophs^*a*^

COG category	Function	apP1	apP2	miP1	miP2	apS1	apS2	miS1	miS2	apD1	apD2	miD1	miD2	apA1	apA2	miA1	miA2
E	Amino acid transport and metabolism	67	67	80	72	81	81	85	72	45	44	42	35	59	58	55	51
S	Function unknown	62	58	69	56	69	71	76	64	34	23	18	24	41	41	42	41
J	Translation, ribosomal structure, and biogenesis	60	60	75	61	91	93	99	87	62	49	46	46	61	59	65	57
C	Energy production and conversion	52	59	57	54	59	57	62	55	42	37	38	34	39	39	43	37
R	General function prediction only	47	45	55	50	59	71	74	58	28	24	27	24	42	43	46	42
M	Cell wall/membrane/envelope biogenesis	40	53	57	43	64	67	71	60	39	32	22	24	35	38	33	34
G	Carbohydrate transport and metabolism	35	31	34	28	49	50	55	47	19	15	14	11	16	14	16	13
O	Posttranslational modification, protein turnover, chaperones	35	36	43	36	52	53	56	47	36	32	27	28	28	26	27	28
P	Inorganic ion transport and metabolism	34	31	35	27	42	41	50	38	30	22	14	18	35	33	38	28
H	Coenzyme transport and metabolism	32	33	39	33	48	50	54	40	21	15	12	10	27	26	25	23
K	Transcription	29	29	34	25	36	36	42	34	26	21	21	21	28	28	29	27
I	Lipid transport and metabolism	26	27	36	28	38	37	40	38	24	19	21	20	31	33	33	31
T	Signal transduction mechanisms	26	24	29	24	31	33	33	33	24	17	17	17	12	16	15	15
L	Replication, recombination, and repair	21	21	31	26	51	54	60	49	19	9	16	13	28	26	30	23
F	Nucleotide transport and metabolism	20	24	23	20	36	35	36	33	18	14	9	13	27	23	26	26
D	Cell cycle control, cell division, chromosome partitioning	16	15	16	11	13	14	14	13	7	4	5	5	14	12	15	11
N	Cell motility	15	15	15	14	18	19	20	12	13	13	6	6	1	0	2	0
V	Defense mechanisms	9	9	10	9	17	17	17	15	8	8	4	5	8	11	10	8
Q	Secondary metabolite biosynthesis, transport and, catabolism	7	6	12	7	7	9	11	5	10	10	4	2	7	7	8	6
U	Intracellular trafficking, secretion, and vesicular transport	7	9	10	9	16	12	16	12	8	7	9	6	6	6	5	5
HR	Coenzyme transport and metabolism	4	4	4	4	4	4	5	3	2	2	2	2	3	3	3	2
	General function prediction only																
TK	Signal transduction mechanisms	4	5	4	4	4	4	3	4	6	5	4	6	3	2	2	3
	Transcription																
NT	Cell motility	4	5	4	4	5	4	5	4	3	2	3	3	2	2	2	2
	Signal transduction mechanisms																
X	Mobilome: prophages, transposons	4	7	4	6	4	5	8	5	4	2	2	3	1	0	0	0
GM	Carbohydrate transport and metabolism	3	1	3	3	3	4	4	4	1	0	1	1	2	1	0	1
	Cell wall/membrane/envelope biogenesis																
KT	Transcription	3	3	5	3	2	2	2	2	3	2	1	0	2	1	1	2
	Signal transduction mechanisms																
EH	Amino acid transport and metabolism	2	3	4	4	4	5	5	4	3	3	2	1	3	4	4	4
	Coenzyme transport and metabolism																
IQ	Lipid transport and metabolism	2	2	3	2	2	2	3	1	2	3	3	2	2	2	2	2
	Secondary metabolite biosynthesis, transport, and catabolism																
MN	Cell wall/membrane/envelope biogenesis	2	2	2	1	1	1	1	1	0	0	0	0	1	1	1	1
	Cell motility																

a mi, Microcystis aeruginosa PCC 7941; ap, Aphanizomenon flos-aquae PCC 7905; P, Pseudomonas putida EK59; S, Stenotrophomonas rhizophila EK20; D, Delftia sp. F45; A, Acinetobacter beijerinckii F107.

Other functional categories involved in cell growth and survival; carbohydrate, coenzyme, and inorganic ion transport and metabolism; protein turnover; and chaperones were also among the most highly expressed genes in all heterotrophs (Table 3). A considerable number of putative defense mechanisms were expressed in most of the heterotrophs, particularly in the S. rhizophila EK20 cocultures. Most of the heterotrophs featured high expression of the CspA family cold shock protein (3% for A. beijerinckii F107 and S. rhizophila EK20, 0.8% for P. putida EK59), known to protect bacterial cells from damage due to low temperature. S. rhizophila featured high expression of 5-formyltetrahydrofolate cyclo-ligase, which is involved in folate metabolism, and the l,d-transpeptidase catalytic domain involved in peptidoglycan cross-linking.

### Highly expressed genes in cyanobacteria.

In both A. flos-aquae PCC 7905 and M. aeruginosa PCC 7941, genes coding for ribosomal proteins and amino acid synthesis, as well as energy production and conversion required for cell growth, were highly expressed (Table 4). Genes involved in cell wall and membrane/envelope biogenesis, protein turnover, chaperone and carbohydrate transport, and metabolism were also equally highly expressed in both cyanobacteria. Several highly expressed genes (19 and 25, respectively) are involved in defense mechanisms and signal transduction (Table S5). Photosystem q(b) protein and Lhc-like protein Lhl4 (3.7 to 5% and 1 to 3.9%, respectively) were the most abundantly expressed genes in the M. aeruginosa PCC 7941 cocultures, while gas vesicle structural protein and allophycocyanin were highly abundant in the A. flos-aquae PCC 7905 cocultures (10 to 12% and 2 to 2.5%, respectively).

**TABLE 4:** Number of highly expressed genes by cyanobacteria

COG category	Function	miA1	miA2	miD1	miD2	miP1	miP2	miS1	miS2	apA1	apA2	apD1	apD2	apP1	apP2	apS1	apS2
J	Translation, ribosomal structure, and biogenesis	106	87	60	64	84	88	108	83	112	107	102	91	87	85	118	121
R	General function prediction only	91	82	54	59	78	78	107	70	113	105	78	80	66	66	118	116
E	Amino acid transport and metabolism	85	69	51	45	63	64	94	68	90	91	68	63	65	53	97	97
H	Coenzyme transport and metabolism	64	52	34	34	56	60	75	42	74	71	57	55	46	43	89	87
S	Function unknown	60	44	29	30	49	42	69	44	80	67	63	52	50	46	91	91
C	Energy production and conversion	58	49	44	42	49	52	60	49	68	64	56	55	49	48	67	67
P	Inorganic ion transport and metabolism	58	42	35	38	35	44	57	44	71	57	52	57	40	42	77	69
M	Cell wall/membrane/envelope biogenesis	54	50	34	39	47	45	61	37	66	67	55	52	47	48	70	69
O	Posttranslational modification, protein turnover, chaperones	50	41	35	38	40	48	55	44	61	61	55	52	48	45	69	66
G	Carbohydrate transport and metabolism	51	41	36	38	47	47	54	42	56	51	53	47	43	40	59	56
L	Replication, recombination, and repair	40	31	26	26	33	32	49	27	60	48	42	38	34	33	60	61
F	Nucleotide transport and metabolism	35	26	23	24	29	30	36	24	36	36	33	26	21	22	43	40
I	Lipid transport and metabolism	24	25	17	17	20	20	26	22	31	26	21	20	22	18	31	31
V	Defense mechanisms	22	21	13	14	20	19	25	16	22	23	20	17	16	15	23	26
T	Signal transduction mechanisms	20	19	15	18	19	19	26	20	31	32	25	26	19	23	35	32
K	Transcription	18	15	9	12	16	15	20	11	26	27	23	19	15	18	33	29
U	Intracellular trafficking, secretion, and vesicular transport	9	10	7	8	10	11	11	9	15	13	13	12	9	7	15	16
D	Cell cycle control, cell division, chromosome partitioning	10	9	6	7	8	9	10	6	14	12	14	11	10	9	15	13
Q	Secondary metabolite biosynthesis, transport, and catabolism	10	8	7	6	9	7	8	8	11	10	11	7	8	6	17	16
X	Mobilome: prophages, transposons	9	6	8	6	8	7	9	8	17	15	14	10	10	12	15	16
IMG/GenBank	Photosynthesis	32	32	18	18	31	31	26	26	18	18	17	17	18	18	18	18
IMG/GenBank	Gas vesicle	3	3	3	3	3	3	3	3	2	2	1	1	1	1	2	2
EH	Amino acid transport and metabolism	6	6	3	4	3	6	6	6	4	4	4	4	4	4	4	4
	Coenzyme transport and metabolism																
NW	Cell motility	4	4	3	4	4	3	4	2	5	6	3	4	5	3	7	8
	Extracellular structures																
HR	Coenzyme transport and metabolism	4	4	2	4	3	4	4	2	6	4	5	4	5	3	6	6
	General function prediction only																
TK	Signal transduction mechanisms	4	3	4	3	3	4	4	4	4	3	3	3	3	3	4	3
	Transcription																
EF	Amino acid transport and metabolism	3	3	1	2	2	3	3	2	3	3	3	3	3	3	3	3
	Nucleotide transport and metabolism																
N	Cell motility	4	3	3	2	3	3	3	2	3	4	2	1	2	3	3	4
NT	Cell motility	2	2	2	1	1	2	3	2	5	4	3	5	2	4	4	5
	Signal transduction mechanisms																
PR	Inorganic ion transport and metabolism	3	3	2	2	2	3	2	2	3	3	3	2	2	2	3	4
	General function prediction only																
HI	Coenzyme transport and metabolism	2	2	1	2	2	1	2	1	3	3	3	3	3	3	3	3
	Lipid transport and metabolism																
EQ	Amino acid transport and metabolism	1	0	1	0	1	0	2	0	0	0	0	1	0	0	1	1
	Secondary metabolite biosynthesis, transport, and catabolism																
IMG/GenBank	Heterocyst differentiation protein	0	0	0	0	0	0	0	0	2	2	2	2	2	2	2	2

a mi, Microcystis aeruginosa PCC 7941; ap, Aphanizomenon flos-aquae PCC 7905; P, Pseudomonas putida EK59; S, Stenotrophomonas rhizophila EK20; D, Delftia sp. F45; A, Acinetobacter beijerinckii F107.

### Differential expression gene profiles in P. putida EK59 and S. rhizophila EK20.

**FIG 3:**
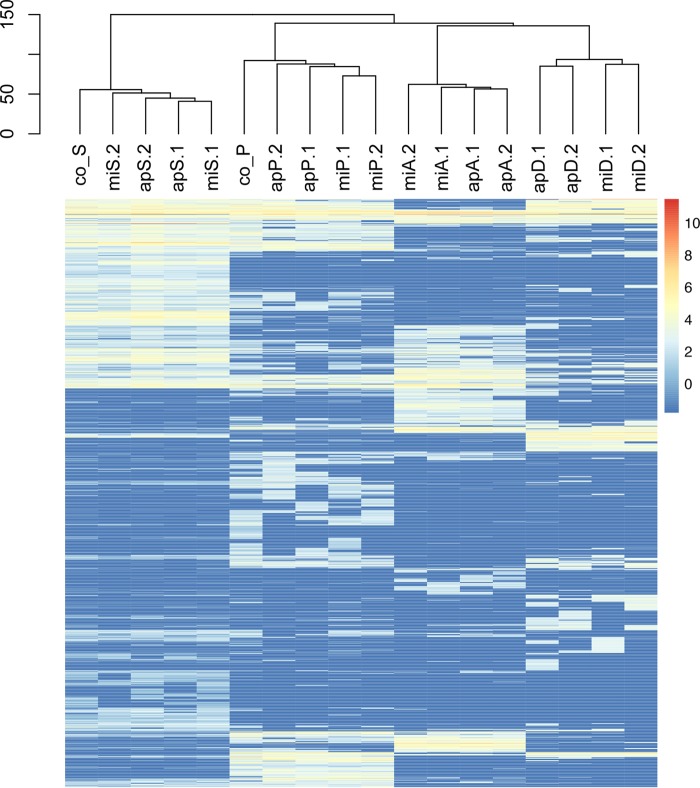
Heatmap displaying the overall expression profiles of heterotrophic bacteria. mi, Microcystis aeruginosa PCC 7941; ap, Aphanizomenon flos-aquae PCC 7905; P, Pseudomonas putida EK59; S, Stenotrophomonas rhizophila EK20; D, Delftia sp. F45; A, Acinetobacter beijerinckii F107; co_S, monoculture control of Stenotrophomonas rhizophila EK20; co_P, monoculture control of Pseudomonas putida EK59. Low transcript abundances are at the blue end of the bar and high transcript abundances are at the red end of the bar.

**FIG 4:**
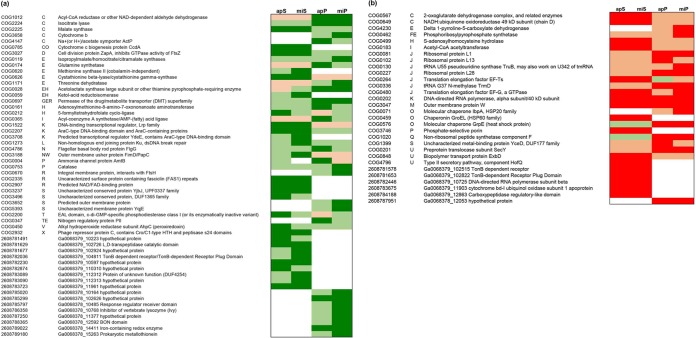
(a and b) Simplified heatmap displaying genes of heterotrophic bacteria that were significantly upregulated (a) or downregulated (b) in at least one coculture compared to the respective monoculture control. The differential expression analyses were performed with the R-package DESeq2 and are based on the comparison of duplicate coculture treatments against one monoculture control treatment. mi, Microcystis aeruginosa PCC 7941; ap, Aphanizomenon flos-aquae PCC 7905; P, Pseudomonas putida EK59; S, Stenotrophomonas rhizophila EK20; D, Delftia sp. F45; A, Acinetobacter beijerinckii F107. Dark green, significantly upregulated; light green, upregulated; dark red, significantly downregulated; light red, downregulated. The following letters explain overall functional COG categories: J, translation, ribosomal structure, and biogenesis; A, RNA processing and modification; K, transcription; L, replication, recombination, and repair; B, chromatin structure and dynamics; D, cell cycle control, cell division, and chromosome partitioning; Y, nuclear structure; V, defense mechanisms; T, signal transduction mechanisms; M, cell wall/membrane/envelope biogenesis; N, cell motility; W, extracellular structures; U, intracellular trafficking, secretion, and vesicular transport; O, posttranslational modification, protein turnover, and chaperones; X, mobilome: prophages, and transposons; C, energy production and conversion; G, carbohydrate transport and metabolism; E, amino acid transport and metabolism; F, nucleotide transport and metabolism; H, coenzyme transport and metabolism; I, lipid transport and metabolism; P, inorganic ion transport and metabolism; Q, secondary metabolite biosynthesis, transport, and catabolism; R, general function prediction only; S, function unknown. CoA, coenzyme A; dsDNA, double-stranded DNA.

Overall clustering of expression profiles of the heterotrophs revealed clear species-specific patterns under all coculture and monoculture conditions. The monoculture control transcriptomes of P. putida EK59 and S. rhizophila EK20 were distinct but clustered with their respective coculture transcriptomes (Fig. 3). Differential expression analyses identified 37 COG homologs and an additional 7 genes annotated as being significantly upregulated in at least one of the coculture treatments (Fig. 4a). Thirty-two COG homologs and 7 annotated genes were downregulated in at least one of the coculture treatments compared to monocultures (Fig. 4b). Expression of some COG homologs was specific to either P. putida EK59 or S. rhizophila EK20, but a few contradictory trends were observed. On the whole, most genes with significant differential expression in one of the cocultures were consistently up- or downregulated across species (Fig. 4a and b).

**FIG 5:**
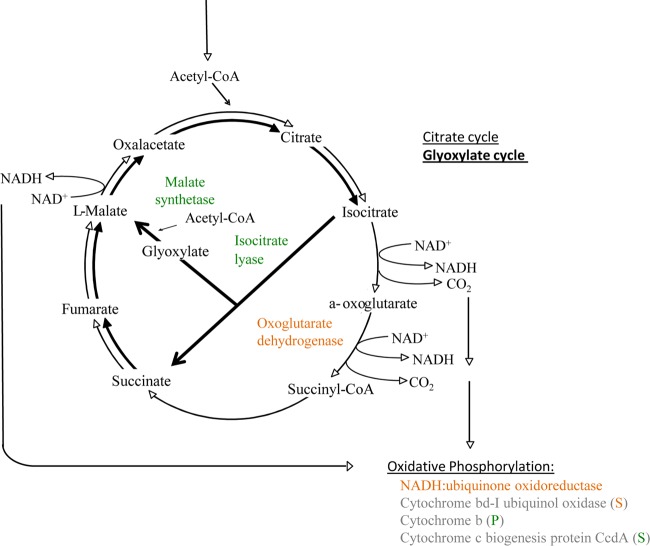
Illustration of the citrate cycle and the glyoxylate shunt (bold). Enzymes that were, according to the differential expression analyses in heterotrophs, significantly differentially expressed in at least one coculture treatment are displayed in green or red, if they were in all treatments (that expressed the gene) up- or downregulated, respectively. In case of inconsistent expression patterns, color-labeled letters (P, P. putida EK59; S, S. rhizophila EK20) indicate in which of the heterotroph strains they were up- or downregulated. (Modified from reference 29 with permission [copyright 2015 Society for Applied Microbiology and John Wiley & Sons Ltd.].)

Among transcripts coding for energy production and conservation, two key enzymes that indicate the activity of the glyoxylate shunt (isocitrate lyase and malate synthetase) were consistently upregulated in cocultures (Fig. 4a). In contrast, a number of other genes coding for enzymes involved in the citrate cycle or reoxidation of reduced NADH_2_ (respiration chain) were consistently downregulated in the cocultures, although only statistically significantly in the S. rhizophila EK20 cocultures (Fig. 4b). Other genes involved in the respiratory chain, such as cytochromes, were inconsistently regulated (Fig. 4a and b). A simplified metabolic map with the transcriptional response of these pathways is given in Fig. 5.

A catalase and alkyl hydroperoxide oxidase protein, as well as a DNA-break repair enzyme, were consistently upregulated in both heterotroph cocultures (Fig. 4a). For both heterotrophs, some transcripts coding for translation and ribosomal structure were consistently downregulated in coculture (Fig. 4b).

Genes involved in formation of flagella were consistently upregulated in both cocultured heterotrophs. Outer or integral membrane and a surface protein containing fasciclin repeats (COG3188, COG0670, and COG2335, respectively) were significantly upregulated, while the outer membrane protein (COG3047) was downregulated in P. putida EK59 cocultures. The 5-formyltetrahydrofolate cyclo-ligase gene involved in folate metabolism was upregulated in most of the cocultured heterotrophs with significant signal in apS.

Among the genes that were significantly upregulated in cocultures was one enzyme with putative extracellular lytic activity. This may have caused the observed cyanolytic activity with l,d-transpeptidase, which was upregulated in S. rhizophila EK20. However, a number of outer membrane candidate genes with potential lytic activities seemed to be upregulated, albeit this effect was not statistically significant. Three outer membrane-coding genes, murein dd-endopeptidase (COG0739), a phospholipase (COG2829), and a penicillin V acylase/amidase (COG3049) (at log_2_ fold changes in miS/apS of 1.9/0.9, 1.3/1.7, and 1.6/1, respectively), were differentially expressed in S. rhizophila EK20 cocultures. In miP, the expression of a muramoyl-tetrapeptide carboxypeptidase (COG1619; log_2_ fold change, 1.1) was increased, while in apP, the expression of an *N*-acetyl muramoyl–l-alanine amidase (COG0860; log_2_ fold change, 1.4) was increased. Murein dd-endopeptidase (score, 9.92) is predicted with a high degree of certainty to be localized in the outer membrane of the bacterial cell and muramoyl-tetrapeptide carboxypeptidase in the cytoplasm (score, 9.26), while the other predicted lytic genes have unknown or multiple localizations in the cell.

### Differential expression gene profile of cyanobacteria.

**FIG 6:**
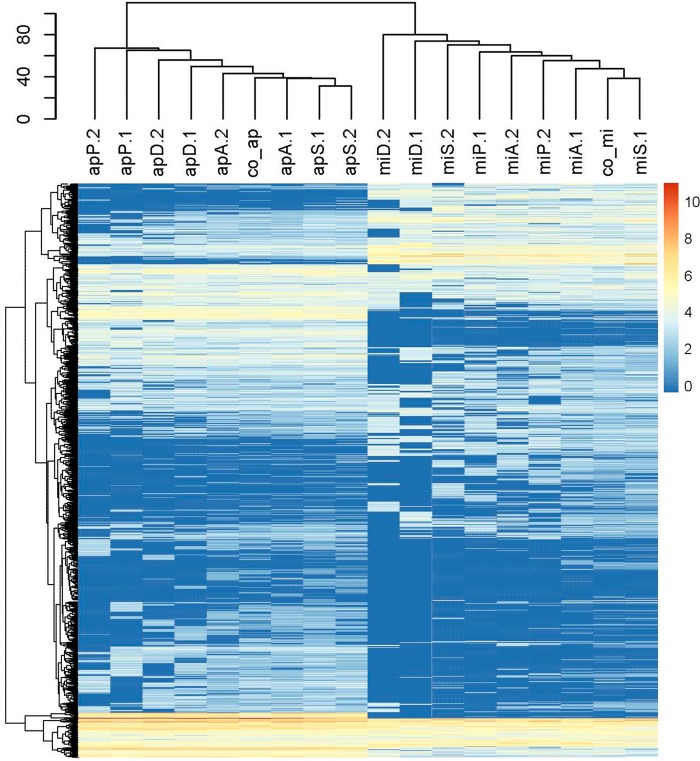
Heatmap displaying the overall expression profiles of cyanobacteria. mi, Microcystis aeruginosa PCC 7941; ap, Aphanizomenon flos-aquae PCC 7905; P, Pseudomonas putida EK59; S, Stenotrophomonas rhizophila EK20; D, Delftia sp. F45; A, Acinetobacter beijerinckii F107; co_mi, monoculture controls of Microcystis aeruginosa PCC 7941; co_ap, monoculture control of Aphanizomenon flos-aquae PCC 7905. Low transcript abundances are at the blue end of the bar, and high transcript abundances are at the red end of the bar.

**FIG 7:**
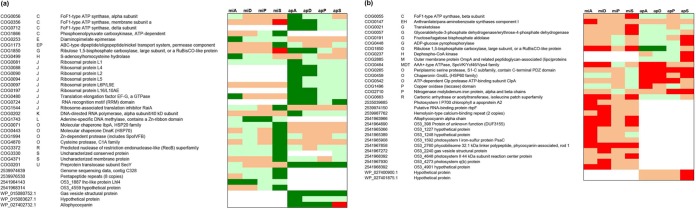
(a and b) Simplified heatmaps displaying genes of cyanobacteria that were significantly upregulated (a) or downregulated (b) in at least one coculture compared to the respective monoculture control. The differential expression analyses were performed with the R package DESeq2 and are based on the comparison of duplicate coculture treatments against one monoculture control treatment. mi, Microcystis aeruginosa PCC 7941; ap, Aphanizomenon flos-aquae PCC 7905; P, Pseudomonas putida EK59; S, Stenotrophomonas rhizophila EK20; D, Delftia sp. F45; A, Acinetobacter beijerinckii F107. Dark green, significantly upexpressed; light green, upregulated; dark red, significantly downregulated; light red, downexpressed. The following letters explain overall functional COG categories: J, translation, ribosomal structure, and biogenesis; A, RNA processing and modification; K, transcription; L, replication, recombination, and repair; B, chromatin structure and dynamics; D, cell cycle control, cell division, and chromosome partitioning; Y, nuclear structure; V, defense mechanisms; T, signal transduction mechanisms; M, cell wall/membrane/envelope biogenesis; N, cell motility; W, extracellular structures; U, intracellular trafficking, secretion, and vesicular transport; O, posttranslational modification, protein turnover, and chaperones; X, mobilome: prophages, and transposons; C, energy production and conversion; G, carbohydrate transport and metabolism; E, amino acid transport and metabolism; F, nucleotide transport and metabolism; H, coenzyme transport and metabolism; I, lipid transport and metabolism; P, inorganic ion transport and metabolism; Q, secondary metabolite biosynthesis, transport, and catabolism; R, general function prediction only; S, function unknown.

Similar to the expression profiles of the heterotrophic organisms, cyanobacteria were also characterized by clear species-specific patterns (Fig. 6). Unlike the heterotrophs, monoculture control treatments did not cluster separately from coculture treatments (Fig. 6). Differential expression analyses revealed 34 genes that were significantly upregulated in at least one of the coculture treatments and 31 genes that were significantly downregulated in at least one of the coculture treatments compared to the monoculture controls (Fig. 7a and b). In contrast to the set of differentially expressed genes among the heterotrophic organisms, the expression profiles in the cyanobacteria responded less consistently to the experimental manipulation treatments (Fig. 7a and b).

Some expression patterns indicated a contrasting physiological response in M. aeruginosa PCC 7941 and *A*. flos-aquae PCC 7905. Several genes involved in photosynthesis were significantly and consistently downregulated in the M. aeruginosa cocultures (photosystem proteins, phycobilisome, and allophycocyanin; Fig. 7b), while the light-harvesting protein Lhl4 was consistently significantly upregulated in miA and miD cocultures (Fig. 7a). In A. flos-aquae, protein-coding genes involved in photosynthesis (F_o_F_1_-type ATP synthase and RuBisCO) were significantly upregulated in apA and apD cocultures, while the same genes were downregulated when these heterotrophs were cocultured with M. aeruginosa (Fig. 7a and b). Such contrasting regulation response was also observed for genes in the COG category translation, ribosomal structure, and biogenesis (Fig. 7a and b). The *A*. flos-aquae gas vesicle structural protein (GenBank accession no. WP_015080752.1) was significantly upregulated in all cocultures involving this organism, while the corresponding gene in M. aeruginosa was significantly downregulated in miD. The nitrogenase molybdenum-iron protein, involved in nitrogen fixation, was downregulated in all *A*. flos-aquae cocultures (statistically significant in two cocultures) and three M. aeruginosa cocultures (Fig. 7b).

## DISCUSSION

In this study, we isolated, cocultured, and analyzed four bacterial antagonists, *S*. rhizophila EK20, P. putida EK59, Delftia sp. F45, and A. beijerinckii F107, with either M. aeruginosa PCC 7941 or *A*. flos-aquae PCC 7905. The type of interaction between different heterotrophs and cyanobacteria in cocultures was revealed by several bacterial responses.

### Heterotrophic responses in coculture.

Outer membrane proteins have been described in Escherichia coli as multifunctional proteins that maintain the integrity of cell shape, diffusion, and release of molecules, while also contributing to virulence by inhibiting the host defense system (21). A few outer membrane proteins that were significantly differentially expressed in our experiments corroborate the microscopic observation of direct physical interactions between heterotrophs and cyanobacteria (Fig. 1, 2, and 4a). Additionally, the consistent upregulation of genes coding for the formation of flagella in S. rhizophila EK20 and P. putida EK59 cocultures suggests cell-to-cell contact antagonism in which heterotrophs appeared to be actively seeking out and attaching to cyanobacterial targets (Fig. 1 and 4a). This is not surprising, as flagellar proteins have previously been linked to virulence, biofilm formation, and adhesion (22). Furthermore, an important role of the fasciclin I domain (FAS1) in cell adhesion has recently been described in Mycobacterium tuberculosis and Rhodobacter sphaeroides (23). This protein-coding gene was significantly upregulated in S. rhizophila EK20 cocultures, suggesting a role in aggregation and biofilm formation on cyanobacterial surfaces (Fig. 1).

Differential expression of several genes that fall into the COG category energy production and conservation (Fig. 4a and b) implies a switch in organic matter processing through the glyoxylate shunt instead of the full Krebs cycle in S. rhizophila EK20 and P. putida EK59 (24, 25). Parameters that have been shown to induce upregulation of the glyoxylate shunt are, for example, the scarcity of high-quality organic matter for cell growth (26) or iron deficiency (27–29). For our study, it seems reasonable to assume that the switch from Casitone-containing growth medium for heterotrophic controls to a medium without such Casitone subsidies may have caused the observed upregulation of the glyoxylate cycle. While the algal exudates are likely to represent high-quality organic substrates in the coculture treatments, the ambient concentration of such substrates in coculture may not be sufficient to satisfy the heterotrophic carbon demand.

Previous work has shown that Shewanella sp. strain W3-18-1 (Shewanella W3-18-1) and Synechococcus sp. strain 7002 (Synechococcus 7002) grown in carbon-limited coculture have an efficient strategy to overcome oxidative stress conditions. This involves an exchange of metabolites and increase in the availability of Fe ions by means of extracellular Fe chelators produced by Shewanella W3-18-1, which can then be acquired by Synechococcus 7002 for metabolic use (30). This might explain the upregulation of the gene coding for 5-formyltetrahydrofolate cyclo-ligase in one of the P. putida EK59 cocultures and all S. rhizophila EK20 cocultures (Fig. 4a). This ligase would facilitate the conversion of 5-formyltetrahydrofolate to 5,10-methenyltetrahydrofolate, a product essential for the metabolism of one-carbon compounds likely released by the cyanobacteria (31, 32). In addition, all four heterotrophs featured high expression of detoxification genes, such as catalase and alkyl hydroperoxide reductase (Table S4). The products of these genes are used for protection from radiation-induced cell damage and oxidative stress and have been shown to be highly expressed in Escherichia coli and Deinococcus radiodurans (33). The high expression of peroxiredoxin, known to protect bacteria from toxic peroxides, was another indication that the heterotrophs suffer from stress (34).

Essential functional categories for growth maintenance, such as ribosomal proteins, translation, and transcription-processing factors, and energy production were highly expressed in all four heterotrophs (35) (Table 3), even though some of these genes were downregulated compared to their respective controls (Fig. 4b). Cyanobacteria play an important role in providing heterotrophic bacteria with newly fixed nitrogen and reduced carbon compounds. For example, a mesocosm experiment in the Baltic Sea showed that isotopically labeled fixed nitrogen from Aphanizomenon was taken up by attached heterotrophic bacteria (36). This implies that the changes in organic matter supply as well as oxygen stress are likely to have a significant impact on the growth of heterotrophs in coculture.

Stenotrophomonas spp. possess a large number of hydrolytic enzymes which act as antibacterial and antifungal compounds by degrading structural and functional proteins of host cells (37–39). Accordingly, the significant upregulation of l,d-transpeptidase in S. rhizophila EK20 possibly disrupts the cross-linkages within the peptidoglycan layer of the cocultured cyanobacteria. In addition, the upregulation of few predicted extracellular hydrolytic genes in both S. rhizophila EK20 and P. putida EK59 indicate that heterotrophs were probably initiating the production of extracellular enzymes. These could be causing the distortion in cyanobacterial cells that was observed microscopically after 96 h of incubation (Fig. 1 and 2) and supports the complete plaque formation seen after 3 to 4 days of incubation (see Materials and Methods).

### Cyanobacterial responses in coculture.

A comparison of the expression patterns in cyanobacterial monoculture controls to those in cocultures identified a small number of genes that were consistently up- or downregulated (Fig. 7a and b). Several photosynthetic genes were downregulated in the M. aeruginosa PCC 7941 cocultures (Fig. 7b), suggesting that photosynthesis was inhibited in this organism in response to the activity of the heterotrophs. It was previously reported that microorganisms minimize energy acquisition and metabolism when exposed to environmental changes (40). In addition, it has been shown that Bacillus mycoides B16 attached to M. aeruginosa in coculture experiments caused a deterioration in light exposure, resulting in the accumulation of glycogen, poly-beta-hydroxybutyrate, and cyanophycin (41). This indicates that either the presence of heterotrophs directly influences photosynthetic activities of the autotroph organisms or that there is an indirect effect by induction of clumps or aggregate formation limiting light exposure (Fig. 1 and S2). The upregulation of the gene for the alternative light-harvesting protein Lhl4 (Fig. 7a) may be a strategy for M. aeruginosa PCC 7941 to maintain basal photosynthetic activity under adverse conditions.

In contrast, A. flos-aquae PCC 7905 cocultures featured an upregulation of photosynthetic genes and genes contributing to gas vesicles which provide regulation of cellular buoyancy. The effect of buoyancy on A. flos-aquae has been studied in the Baltic Sea, where a 2-fold increase in photosynthesis was observed in response to wind-induced mixing events (42). Moreover, the gene coding for the nitrogenase molybdenum-iron protein, the central gene of nitrogen fixation, was consistently downregulated in all A. flos-aquae PCC 7905 cocultures. This implies that the attachment of S. rhizophila EK20 and P. putida EK59 to heterocysts might have an effect on nitrogen fixation-coding genes in the A. flos-aquae PCC 7905 cocultures. However, the same photosynthesis-coding genes were significantly upregulated in A. flos-aquae PCC 7905-Delftia sp. F45 and A. beijerinckii F107 coculture, even if they were not predominantly attached to heterocysts. This indicates that there is a specific response of A. flos-aquae PCC 7905 to different heterotrophic strains, or it may be that Delftia sp. F45 and A. beijerinckii F107 had a weaker interaction with the photosynthetic cells, at least compared to that with S. rhizophila EK20 and P. putida EK59.

In keeping with the four cocultured heterotrophs, the cyanobacteria also featured high expression of growth-related genes, such as ribosomal proteins, amino acid and coenzyme synthesis, and energy production by the Krebs cycle, for maintenance of growth (Table S5). This suggests that cyanobacteria were actively growing without being critically starved for energy or nutrients.

The cyanobacterial model organism Synechocystis sp. strain PCC 6803 harbors 47 pairs of type II toxin-antitoxin systems (TA systems) that exhibit RNase activity and which may have other additional undiscovered functions (43). The production of antitoxin components is usually induced by stress responses linked to reversible growth inhibition or cell death. For example, in order to adapt to environmental stresses via reversible growth arrest, Anabaena sp. strain PCC 7120 expressed genes for the chromosomal type II toxin-antitoxin systems (44). Both of our studied cyanobacterial strains also expressed high levels of several antitoxin components, likely representing defense mechanisms to maintain their growth under stress conditions (Tables 4 and S5).

### Conclusions.

Our study provides new information on the biology of heterotrophic bacteria capable of lysing cyanobacteria and, in doing so, identifies diverse physiological traits that likely shape these interactions. The overall transcriptional responses of heterotrophs in coculture with cyanobacteria indicate that their interaction could be divided into four main categories: (i) cell-to-cell contact, (ii) nutrient and space competition with cyanobacteria, (iii) entrapment of cyanobacteria with aggregate formation, and (iv) production of extracellular compounds to disrupt or damage cyanobacterial cells. In contrast, heterotroph aggregate formation interferes with M. aeruginosa PCC 7941 photosynthetic processes, and the preferential attachment of the different heterotrophs to either heterocyst or photosynthetic cells of A. flos-aquae PCC 7905 initiates contrasting antagonistic gene expression related to nitrogen fixation and photosynthesis processes. We conclude that the metatranscriptome analysis of samples collected after 24 h of a coculture experiment was only a snapshot of the antagonistic interaction and did not capture the complete mechanism underlying the antagonistic interaction.

## MATERIALS AND METHODS

### Cyanobacterial cultures.

Two cyanobacterial strains, Microcystis aeruginosa PCC 7941 and Aphanizomenon flos-aquae PCC 7905, were obtained from the Pasteur Culture Collection as experimental model strains for lytic tests. M. aeruginosa PCC 7941 is a unicellular, planktonic, and toxin-producing strain capable of buoyancy regulation with gas vesicles. A. flos-aquae PCC 7905 is a filamentous and toxin-producing strain with the ability to fix nitrogen. Both strains represent idealized models of bloom-forming freshwater cyanobacteria of environmental concern. Both strains were cultured and maintained in a cyanobacterial BG11 freshwater medium (45) at 20°C under photosynthetically active radiation (PAR) of approximately 5 μE m^2^ s^−1^ (IL-1400 radiometer with PAR sensor), with a 12-h light/12-h dark cycle.

### Isolation of cyanolytic bacteria.

Water samples from three eutrophic lakes in south-central Sweden, Erken, Ekoln, and Funbosjön, were collected during a summer period of high cyanobacterial biomass to screen for cyanolytic bacteria. Lytic bacterial strains were isolated by first preparing a cyanobacterial lawn according to the method of Whyte et al. (46). A volume of 15 ml of each of the axenic cyanobacterial cultures (PCC 7941 and PCC 7905) at cell densities of approximately 4.8 × 10^5^ cells · ml^−1^ was mixed with 0.5 ml of unfiltered lake water. The mixed water samples were then gently filtered onto 0.22-μm-pore-size polyether sulfonate membranes (47 mm diameter; Gelman Supor). Filters were rapidly placed on top of BG11 agarose plates in individual petri dishes, sealed with Parafilm, and incubated under the same light and temperature conditions described above. Viral and bacterial plaque formation was monitored for 3 to 4 days using a stereo microscope (Olympus SZ61) (Fig. S1). Bacterial colonies that formed lytic zones in the cyanobacterial lawns were further purified by repeated streaking onto BG11 agar supplemented with 0.2% Casitone.

### Lytic test.

One hundred bacterial isolates were recovered from the cyanobacterial lawns. In order to confirm that the colonies maintained their lytic activity, we regrew them in BG11 liquid medium supplemented with 0.2% Casitone at 25°C for 48 h and then inoculated 10 to 20 μl of pure culture suspension in triplicate onto a cyanobacterial lawn. The cyanobacterial lawns were photographed after 24 to 72 h of incubation (Canon G9 zoom lens 6 × 15 fixed to a Kaiser RS1 camera stand), and both the colony diameter and the diameter of the lytic halo were quantified using Image J (public domain image processing and analysis in Java). The enzymatic activity was then reported as the colony diameter divided by the halo diameter ratio, expressed as the enzymatic index (EI) (47).

### 16S rRNA sequencing.

Thirty-one pure colonies possessing lytic activity were chosen for 16S rRNA gene sequencing. DNA was extracted using the PowerSoil DNA extraction kit (Mo Bio Laboratories, Inc., CA, USA). PCR primers 341F (5′-CCTACGGGNGGCWGCAG-3′) and 805R (5′-GACTACHVGGGTATCTAATCC-3′) were used for 16S rRNA amplification. PCR was conducted in a 20-μl volume using 1 U of *Taq* DNA polymerase (New England BioLabs), 0.25 μM primers, 200 μM dinucleoside triphosphate (dNTP) mix, 0.4 μg of bovine serum albumin, and 1 μl of DNA template. The thermal program consisted of an initial 95°C denaturation step for 5 min, a cycling program of 95°C for 40 s, 53°C for 40 s, and 72°C for 60 s, and a final elongation step at 72°C for 7 min for 20 cycles. Amplicons were purified with a Qiagen gel purification kit (Qiagen, Germany) and quantified with a fluorescent stain-based kit (PicoGreen; Invitrogen). BigDye Terminator version 3.1 (Applied Biosystems, Paisley, UK); primer 341F was used for sequencing reactions, and the samples were subsequently analyzed by capillary electrophoresis on an ABI3730XL DNA analyzer (Applied Biosystems).

### Antagonistic interaction between cyanobacteria and heterotrophic bacterial strains.

Four phylogenetically diverse heterotrophic strains with strong and reproducible cyanolytic activity were selected for further experiments: Stenotrophomonas rhizophila EK20, Pseudomonas putida EK59, Delftia sp. F45, and Acinetobacter beijerinckii F107. Prior to performing coculture experiments, the viability and purity of heterotrophs were verified by inoculation in LB agar plates and by microscopic observation. Inoculation in Luria broth was also used to verify that the cyanobacterial cultures were axenic. Heterotrophic bacteria were quantified by flow cytometry (CyFlow space; Partec, Münster, Germany) after mixing the sample with a 1.25 μM final concentration of SYTO13 nucleic acid stain (Invitrogen, Eugene, OR, USA). The sample flow rate was 4 μl/s, and the sheath fluid was Milli-Q water. SYTO13-stained cells were excited by blue laser (488 nm) with gain setting of 335 nm for side scatter (SSC), 450 for green fluorescent light (FL), and 240 for forward-scatter light (FSC). Bacterial cells were identified according to their FSC and green fluorescence patterns. The average relative cell size was estimated using the mean FSC value and referred as individual cell size (ICS). Biovolume (BV) of the samples was calculated by multiplying ICS with respective bacterial abundance (BA) (48). Cyanobacterial abundance was assessed by fluorescence microscopy counts. The initial ratio of heterotrophs to cyanobacteria in the cocultures was set to approximately 1:1. Each heterotrophic strain was cultured in darkness using liquid BG11 medium supplemented with 0.2% Casitone at 25°C. Cells were harvested after 48 h of incubation by centrifugation (10,000 rpm, 15 min, 25°C) and washed and suspended in Casitone-free BG11 medium before mixing with M. aeruginosa PC 7941 or A. flos-aquae PCC 7905. Cocultures were maintained under photosynthetically active radiation (PAR) conditions of approximately 5 μE m^2^ s^−1^ (IL-1400 radiometer with PAR sensor) with a 12-h light/dark cycle. One milliliter of sample for cell counts was collected from each culture after 6, 24, and 96 h of incubation and fixed with 2% formaldehyde. Additionally, 25 ml of culture was collected by rapid filtration onto 0.2-μm-pore-size Supor membrane filters (47 mm diameter; Pall Corporation) and was immediately frozen at −80°C for later RNA isolation. Control samples of M. aeruginosa PCC 7941 and A. flos-aquae PCC 7905 were collected separately under the same light conditions mentioned above. Heterotrophic controls for *S*. rhizophila EK20 and P. putida EK59 were grown in darkness at 25°C in liquid BG11 medium supplemented with 0.2% Casitone and were collected after 48 h of incubation.

### Epifluorescence microscopy.

Formaldehyde-preserved samples were filtered onto black polycarbonate membrane filters (0.22-μm-pore-size, 25 mm) placed on top of a support filter to ensure even dispersion of cells across the filter area. 4′,6-Diamidino-2-phenylindole was used for DNA staining at a final concentration of 100 μg/ml, whereas NanoOrange (Life Technologies) was used for protein staining at 5.0 μg/ml, according to the manufacturer's instructions. Filters were incubated for 15 min before excess stain was removed by vacuum filtration and further rinsed with deionized water. Image acquisition for each filter section was done using an AxioPlan II epifluorescence microscope with a digital camera (AxioCam) installed (Carl Zeiss, Germany). Cell concentrations of both heterotrophs and cyanobacteria were calculated from the equation (*C* × *A_F_*)/(*n* × *V* × *A_G_*), where *C* is the total number of bacteria counted, *A_F_* is the effective area of the membrane filter, *A_G_* is the area of the observed grid, *n* is the number of grids counted, and *V* is the volume of sample filtered. Two sample replicates were counted, with the number of counted grids ranging from 8 to 15 grids per sample. Rough counts of normal heterotrophs and cyanobacterial cell shapes were counted due to the formation of heterotrophic aggregates after 24 h of incubation.

### RNA isolation and sequencing.

Two biological replicates for each combination of the four heterotrophic strains and the two cyanobacteria (M. aeruginosa PCC 7941 or *A*. flos-aquae PCC 7905) were selected for total RNA isolation and metatranscriptome sequencing. Metatranscriptome analyses were carried out for samples retrieved after 24 h of incubation. RNA extraction was performed with the PowerWater RNA isolation kit, as per the manufacturer (Mo Bio Laboratories, USA). Digestion of contaminating DNA was carried out using the Turbo DNA-free kit (Invitrogen, Life Technologies, Europe BV) and the absence of DNA verified by negative PCR amplification of the 16S rRNA gene. RNA integrity was assessed on an Agilent 2100 Bioanalyzer (Agilent Technologies, Inc., USA). First-strand cDNA was synthesized by RevertAid H Minus first-strand cDNA synthesis kit (Thermo Scientific, USA), followed by second-strand cDNA synthesis using a double-stranded DNA synthesis kit (catalog no. E6111; New England BioLabs). The resulting double-stranded DNA was purified by QIAquick PCR extraction kit (Qiagen) and the concentration was measured using the PicoGreen double-stranded DNA assay (Invitrogen). Sequencing was performed on each of two replicate coculture and control samples of Stenotrophomonas rhizophila EK20, Pseudomonas putida EK59, M. aeruginosa PCC 7941, and, A. flos-aquae PCC 7905 (SNP&SEQ Technology Platform at Uppsala University). Libraries were prepared from 20 ng of cDNA using the ThruPLEX FD library preparation kit (Rubicon Genomics) and used for two full runs on the Illumina MiSeq instrument running in paired-end 2 × 300-bp mode with version 3 chemistry.

### Bacterial reference genomes.

The draft genomes of the four selected heterotrophic strains (*S*. rhizophila EK20, P. putida EK59, Delftia sp. F45, and A. beijerinckii F107) were sequenced by MiSeq using 2 × 300 chemistry, as described above. Reads were first assembled using the SPAdes genome assembler, with contigs ≥2,000 bp length with high coverage (>190×) submitted to Integrate Microbial Genomes (IMG) for genome annotation using the IMG pipeline (49). The IMG taxon identification (ID) of each strain is as follows: Delftia sp. F45, Gp0111381; A. beijerinckii F107, Gp0111397; *S*. rhizophila EK20, Gp0111470; and P. putida EK59, Gp0111469.

### RNA processing.

RNA reads were assembled using the de novo assembler Trinity (50), according to the assembly protocol described by Hass et al. (51). The de novo transcriptome assembly was performed by the three constituent components: Inchworm, which assembles unique sequences to construct transcripts; Chrysalis, which makes groups of transcript contigs form components and generate de Bruijn graphs for each component; and Butterfly, which compacts and extracts all probable sequences from each graph. Prior to the mapping of raw RNA reads to the assembly, they were quality trimmed using Sickle (https://github.com/najoshi/sickle) (quality cutoff, 20; minimum read length, 75 bp), and noncoding RNA was removed via the SortMeRNA software (52). The trimmed paired reads were mapped on the assembled Trinity contigs using the Bowtie 2 aligner (53). BLASTP was used to align and annotate Trinity-assembled contigs to the reference genome amino acid sequences of the 4 heterotrophic bacteria (described above) and a group of Microcystis genomes (M. aeruginosa PCC 7041 M. aeruginosa DIANCHI905, M. aeruginosa TAIHU98, M. aeruginosa PCC 9432, M. aeruginosa PCC 9432, M. aeruginosa PCC 9717, and M. aeruginosa PCC 9808), as well as the *A*. flos-aquae NIES-81 genome (BioProject accession no. PRJNA232534). The BLASTP E value cutoff used was e^−2^. While the genome data provided by IMG are annotated to COG homologs, this is not the case for genome data stored in GenBank. Therefore, a BLASTP search of *A*. flos-aquae NIES-81 protein data against the COG reference database was performed with the same E value cutoff provided by other COG annotations (E <10^−2^). RNA reads mapping to the respective reference genome were binned and either assigned to a COG homolog or, if this information was not available, to the overall functional annotation provided by IMG/GenBank for each metagenomic open reading frame. An overview containing the number of obtained reads for each sample is available in Table S1. The localization of lytic genes was predicted by the localization prediction tool PSORTb (http://www.psort.org/psortb/).

### Estimation of highly expressed genes.

Highly expressed genes were extracted from the relative abundance counts (derived after normalization of the raw count data to the contig length) of the expressed genes in heterotrophs and cyanobacteria. For each replicate pair, we sorted genes by their normalized expression values based on the lower value of both replicates in each gene, thus ranking genes high only if expression was high in both replicates. Highly expressed genes were subsequently sorted by COG functional category. While genes with an expression level of 0 were excluded, this does not mean that there is no expression.

### Differential expression analyses.

The transcripts of each strain grown in coculture were compared to their respective control treatment transcripts using a differential expression analysis performed in the R environment with the package DESeq2 (54). For the two heterotrophic bacteria without a heterotroph-only control, no differential expression analyses were performed. *P* values were corrected for multiple testing according to the procedure of Benjamini and Hochberg (55). Transcribed gene orthologs with a corrected *P* value (adjusted) of <0.1 were classed as significantly differentially transcribed genes.

### Accession number(s).

Raw sequence data of the four genomes were submitted to the NCBI database with the following accession numbers: PRJNA310594 for Delftia sp. F45, PRJNA310595 for A. beijerinckii F107, PRJNA310596 for P. putida EK59, and PRJNA310597 for S. rhizophila EK20. RNA reads were submitted to the Bioinformatics Infrastructure for Life Sciences (BILS), and an active doi image was established at https://doi.org/10.17044/BILS/MG00001.

## Supplementary Material

Supplemental material
