# Supplementary material for: Interactions of Freshwater Cyanobacteria with Bacterial Antagonists
Source: Appl Environ Microbiol. 2017 Mar 17;83(7):e02634-16. doi: 10.1128/AEM.02634-16 (PMC5359482; doi:10.1128/AEM.02634-16)
Supplement: Supplemental material [file supp_83_7_e02634-16__index.html]

Supplemental material 

# Interactions of Freshwater Cyanobacteria with Bacterial Antagonists

## Supplemental material

- Supplemental file 1 -

  Number of obtained metatranscriptome reads after sequencing, quality trimming, and sorting out protein coding sequences (Table S1); list of isolated cyanolytic bacteria from cyanobacterial lawn (Table S2); cell counts of heterotrophs and cyanobacteria in coculture replicates (Table S3); representative genes of COG category in heterotrophs (Table S4); representative highly expressed genes in cyanobacteria (Table S5); plaque formation on a *Microcystis aeruginosa* PCC 7941 lawn (Fig. S1); change in *M. aeruginosa* PCC 7941 coculture appearance when exposed to a heterotrophic strain (Fig. S2).

  PDF, 848K
