# Supplementary material for: Interactions of Freshwater Cyanobacteria with Bacterial Antagonists
Source: Appl Environ Microbiol. 2017 Mar 17;83(7):e02634-16. doi: 10.1128/AEM.02634-16 (PMC5359482; doi:10.1128/AEM.02634-16)
Supplement: Supplemental material [file AEM.02634-16_zam999117739s1.pdf]

Table S1. Number of obtained metatranscriptome reads after sequencing, quality trimming and sorting out protein coding sequences. Non-integers in the column for mapped reads indicate that only one of the two paired-ends in at least one read could be mapped to a contig

sample	#nr of reads	#nr of trimmed reads	#nr of protein coding reads	#nr of mapped reads
apA.1	1,812,095	1,802,357	64,785	56,427
apA.2	1,824,545	1,805,568	48,934	42,934.5
apD.1	1,952,648	1,941,888	33,239	24,177
apD.2	1,610,510	1,592,782	26,701	20,223.5
apP.1	1,667,894	1,656,689	19,062	16,375.5
apP.2	1,581,431	1,560,024	19,617	17,224
apS.1	2,102,349	2,090,010	90,516	81,796.5
apS.2	1,641,374	1,621,212	75,289	69,599
miA.1	1,736,382	1,727,589	49,431	44,089
miA.2	1,496,947	1,480,189	30,966	28,666.5
miD1	1,846,184	1,836,308	19,026	15,189
miD2	1,410,130	1,394,928	14,039	11,545
miP1	1,939,166	1,923,857	31,887	28,272
miP2	1,552,316	1,533,835	26,467	24,309
miS1	3,911,211	3,886,741	110,723	103,446.5
miS2	1,502,738	1,484,599	40,242	37,734.5
co_ap	1,753,932	1,737,207	54,083	44,585.5
co_mi	1,646,696	1,626,903	87,478	81,041
co_P	2,106,102	2,079,128	24,935	22,339
co_S	1,487,810	1,469,564	37,456	34,010

Table S2. List of isolated cyanolytic bacteria from cyanobacterial lawn. ER: Erken lake, EK: Ekoln lake, F: Funbojon; EI: Enzymatic Index was calculated by the ratio of colony diameter/halo diameter.

**Lake**	**Strain identity (99-100%)**	**Colony diameter (mean) mm**	**Halo diameter (mean) mm**	**EI mm**	**Halo shape**
ER1	*Pseudomonas* sp.R1SpM3P2C2	50.80	31.38	1.62	defined
ER2	*Pseudomonas* sp. IW-223	39.45	37.66	1.05	defined
ER4	*Pseudomonas* sp. IW-223	31.34	11.85	2.65	defined
ER7	*Acidovorax* sp. SA3	42.11	88.57	0.48	diffuse
ER8	*Pseudomonas* sp. IW-223	39.05	45.98	0.85	defined
ER9	*Pseudomonas migulae*	20.60	74.05	0.28	diffuse
ER10	*Acidovorax* sp. SA3	23.37	65.19	0.36	defined
ER11	*Pseudomonas* sp. PM470	71.22	39.34	1.81	defined
EK18	*Pseudomonas fluorescens* strain PS7	54.63	40.65	1.34	defined
EK20	*Stenotrophomonas* sp. P 16S	51.83	99.94	0.52	diffuse
EK22	Pseudomonas sp.	48.87	86.33	0.57	diffuse
EK23	*Pseudomonas arsenicoxydans*	47.23	89.23	0.53	diffuse
EK24	Pseudomonas fluorescens strain PS7	10.72	10.72	1.00	diffused
EK25	*Marinobacter arcticus* strain T2	15.78	53.84	0.29	diffused
EK59	*Pseudomonas putida*	9.84	26.02	0.38	diffused
F36	*Pseudomonas salomonii*	17.47	14.21	1.23	diffused
F39	*Pseudomonas* sp. R3(2013)	22.75	27.75	0.82	diffused
F40	*Pseudomonas* sp. b21	16.37	17.27	0.95	diffused
F41	*Pseudomonas* libanensis	10.87	27.57	0.39	diffused
F42	*Pseudomonas* sp. R1SpM3P2C2	14.29	31.98	0.45	diffused
F45	*Delftia* sp. M_Sw_oHS_08/10_2_1	11.69	28.98	0.40	defined
ER51	*Pseudomonas* sp. R1SpM3P2C2	20.65	15.83	1.30	defined
ER59	*Pseudomonas putida*	23.68	10.04	2.36	defined
EK68	*Pseudomonas* sp. R3(2013)	15.91	28.00	0.57	defined
EK69	*Pseudomonas fluorescens* strain PS7	14.80	24.40	0.61	defined
F106	*Acinetobacter* sp. JB54	11.25	23.41	0.48	defined
F107	*Acinetobacter beijerinckii*	6.92	5.74	1.21	defined
EK47	*Xanthomonas translucens* strain AS2-61a	8.49	62.86	0.14	diffused
EK50	*Stenotrophomonas maltophilia* strain 261ZG10	15.32	48.95	0.31	diffused
EK56	*Stenotrophomonas* sp.	17.06	21.41	0.80	defined
EK66	*Stenotrophomonas* sp.	22.16	52.97	0.42	diffused

Table S3: Cell count of heterotrophs and cyanobacteria in coculture replicates. Mi: *Microcystis aeurignosa* PCC 7941, Ap: *Aphanizomenon flos-aquae* PCC7905, P: *Pseudomonas putida* EK59, S: *Stenotrophomonas rhizophila* EK20, D: *Delftia* sp. F45, A: *Acinetobacter beijerinckii* F107.

					Number of cells x 10^5 per mL†			
Incubation time (h)	**Mi1**	**P1**	**Mi2**	**P2**	**Ap1**	**P1**	**Ap2**	**P2**
6	10.42	29.17	9.76	29.62	7.50	13.75	3.18	68.18
24	8.75	27.08	10.00	29.62	5.56	55.00	3.02	89.17
96	7.50	31.43	7.92	41.67	5.00	80.45	2.91	93.18
	**Mi1**	**D1**	**Mi2**	**D2**	**Ap1**	**D1**	**Ap2**	**D2**
6	9.62	37.31	2.00	28.50	9.09	27.27	2.50	12.00
24	8.67	39.00	5.38	13.46	5.00	13.33	2.00	58.50
96	9.64	54.29	4.91	25.50	5.00	55.50	1.50	67.50
	**Mi1**	**A1**	**Mi2**	**A2**	**Ap1**	**A1**	**Ap2**	**A2**
6	11.67	26.67	16.67	35.78	5.00	52.27	6.70	47.31
24	10.00	80.56	13.00	108.80	5.83	55.63	10.00	53.89
96	16.11	73.89	17.00	156.98	5.00	61.67	9.50	58.11
	**Mi1**	**S1**	**Mi2**	**S2**	**Ap1**	**S1**	**Ap2**	**S2**
6	10.00	52.27	12.14	137.82	5.00	101.54	5.90	97.55
24	9.09	109.55	15.71	226.25	5.00	154.62	6.54	196.88
96	10.00	137.86	11.42	305.80	10.54	180.46	11.15	334.86

† Aggregates of heterotrophs and irregular shapes of cyanobacterial cells did not allow precise counting after 24 h of incubation

Table S4. Representative genes of COG category in heterotrophs

COG category	Gene description
E	Aminopeptidase,deaminase,lipase, amino synthesis genes
	cold shock protein AA, ABC transporter, amino acid transporter
S	Outer membrane, uncharacterized proteins, Osmotically-inducible protein osmY (MD)
J	Ribosomal proteins L20,L31, S1, L28,S21,S10,L1,L13, translation initiation and elongation factor
C	isocitrate lyase, cytochrome, malate/lactate dehydrogenase,malic enzyme,
	NADH:ubiquinone oxidoreductase, citrate synthase
R	metal dependent hydrolase, protease, metalloprotease,carboxypeptidase,OsmC-related protein
M	Membrane-bound lytic murein transglycosylase B,Cell wall-associated hydrolase,
	Outer membrane protein OmpA,Membrane carboxypeptidase/penicillin-binding protein
G	pyruvate kinase, phosphoglucomutase, transketolase,
	glucose dehydrogenase,glyceraldehyde-3-phosphate dehydrogenase
O	heat shock protein, different types of proteases and chaperone, peroxiredoxin
P	catalase, different type of transport systems,Superoxide dismutase
H	hydrolase, S-adenosylhomocysteine hydrolase, dihydropteroate synthase,GTP cyclohydrolase II,
	Lipoate synthase,Demethoxyubiquinone hydroxylase, Biotin synthase or related enzyme
K	DNA-binding transcriptional regulator
I	Acyl-CoA hydrolase,Fatty-acid desaturase,Acetyl esterase/lipase
T	several Signal transduction histidine kinase,chemotaxis, stress protein,sensory domains
L	glycolyase, DNA polymerase, RecG-like helicase,5'-3' exonuclease,DNA repair photolyase
F	Nucleoside permease NupC,Uridylate kinase,GMP synthase
D	cell division proteins, cell shape determining protein
N	Flagellar biosynthesis proteins, flagellar motor component, flagellar basal body protein
V	Multidrug effluc pump, Beta-lactamase class A, Multidrug resistance efflux pump,Antitoxin component
	Organic hydroperoxide reductase OsmC/OhrA,Type I restriction-modification system,Alkyl hydroperoxide reductase subunit AhpC
	ABC-type multidrug transport system, ATPase component,(peroxiredoxin)
Q	Dienelactone hydrolase,Aldo/keto reductase,Acyl-homoserine lactone (AHL) acylase PvdQ,Enhancing lycopene biosynthesis protein 2
U	secretory pathway components,Preprotein translocase subunit SecB,
	ABC-type protease/lipase transport system,Biopolymer transport protein ExbD,

Table S5. Representative highly expressed genes in cyanobacteria

COG category	Gene description
J	Ribosomal protein L19,S14,S21, S11, L28, RNase P protein component, translation elongation factor,Peptide deformylase,methionine aminopeptidase,aspartyl-tRNA synthetase
R	Predicted Zn-dependent peptidase,Hemolysin or related protein,Endonuclease, Uma2 family, Predicted amidohydrolase,Predicted nuclease of restriction endonuclease-like (RecB)
E	Amino acid synthase,ABC-type branched-chain amino acid transport system, Proline dehydrogenase,Zn-dependent oligopeptidase,Alanine dehydrogenase,Glutaminase
H	Cobalamin biosynthesis protein CbiD,Mg-chelatase subunit ChlI,Lipoate synthase, Coproporphyrinogen III oxidase,Quinolinate synthase,FAD synthase
S	Uncharacterized conserved proteins,Uncharacterized integral membrane protein,Predicted metal-dependent hydrolase
C	Ferredoxin, FoF1-type ATP synthase, Citrate synthase, Malic enzyme, NADH:ubiquinone oxidoreductase 20 kD subunit, Phosphoenolpyruvate carboxylase,Fumarate hydratase class II,Malate/lactate dehydrogenase
P	Nitrogenase molybdenum-iron protein,Cation transport ATPase,High-affinity Fe2+/Pb2+ permease,Catalase,Superoxide dismutase ABC-type nitrate/sulfonate/bicarbonate transport system,Nitrogenase subunit NifH,Ca2+/H+ antiporter,
M	Outer membrane protein TolC,Anionic cell wall polymer biosynthesis enzyme,Cell wall-associated hydrolase Membrane carboxypeptidase (penicillin-binding protein),Membrane-bound lytic murein transglycosylase, Peptidoglycan hydrolase (amidase) enhancer domain,Periplasmic protein TonB, Capsular polysaccharide biosynthesis protein
O	Different types of proteases and chaperones,Ni2+-binding GTPase,Peroxiredoxin,Heat shock protein HslJ,Glutaredoxin-related protein
G	Glucokinase,Pyruvate kinase,Transketolase,Fructose/tagatose bisphosphate aldolase,Glycosidase, Fructose-1,6-bisphosphatase,Glycogen synthase,Transaldolase,Glyceraldehyde-3-phosphate dehydrogenas
L	Ribonuclease HII,DNA repair ATPase RecN,Endonuclease III,DNA gyrase/topoisomerase IV,Uracil-DNA glycosylase
F	Uridine kinase, Guanylate kinase,Adenylosuccinate synthase,Nucleoside permease NupC,Deoxycytidylate deaminase
I	Long-chain acyl-CoA synthetase,Fatty-acid desaturase,Phytoene/squalene synthetase,Biotin carboxylase
V	Antitoxin component HigA of the HigAB toxin-antitoxin module,ABC-type multidrug transport system,toxin component of the YafQ-DinJ toxin-antitoxin module Type I restriction-modification system,Beta-lactamase class A,gas vesicle structural protein,Antitoxin component of toxin-antitoxin stability system, ABC-type multidrug transport system,Tpump,Alkyl hydroperoxide reductase subunit AhpC (peroxiredoxin),Multidrug resistance efflux
T	Signal transduction histidine kinase,Anti-sigma regulatory factor,Ser/Thr protein kinase RdoA involved in Cpx stress response, Serine/threonine protein phosphatase PrpC,K+-sensing histidine kinase KdpD,Nucleotide-binding universal stress protein
K	Exoribonuclease R, Leucine-rich repeat (LRR) protein,Predicted transcriptional regulator,DNA-directed RNA polymerase, Curved DNA-binding protein CbpA,Phage shock protein A,Transcription termination factor NusB, transcriptional regulator of heat shock response
U	Preprotein translocase subunit SecY,Biopolymer transport protein ExbB/TolQ,Signal peptidase I, Type II and IV secretory pathway,Autotransporter translocation and assembly factor TamB
D	Cell division protein FtsN,Spore germination protein GerM,Spore maturation protein CgeB, Septum formation topological specificity factor MinE,Stage III sporulation protein SpoIIIAA
Q	2-keto-4-pentenoate hydratase,Acyl transferase domain in polyketide synthase (PKS) enzyme, Carotenoid cleavage dioxygenase or a related enzyme,Ca2+-binding protein, RTX toxin-related
X	Transposase,Phage-related protein,REP element-mobilizing transposase RayT,Phage tail sheath protein F
IMG/ genebank	Photosystem I reaction center subunit IX ,photosystem I reaction center subunits, phycocyanobilin photosystem II reaction center proteins,allophycocyanin,photosystem II CP47 chlorophyll apoprotein

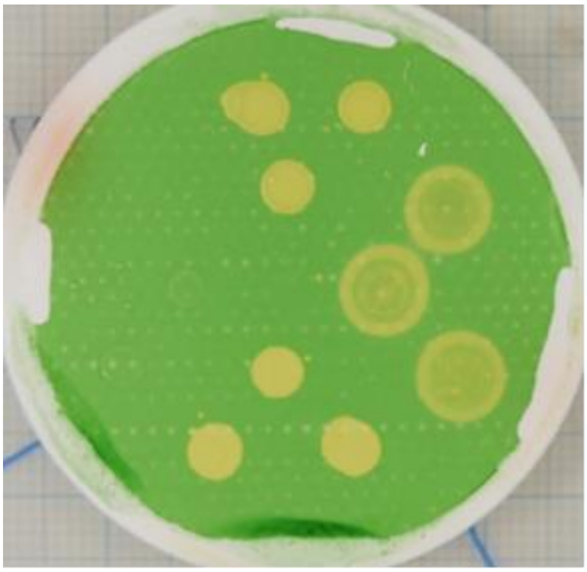

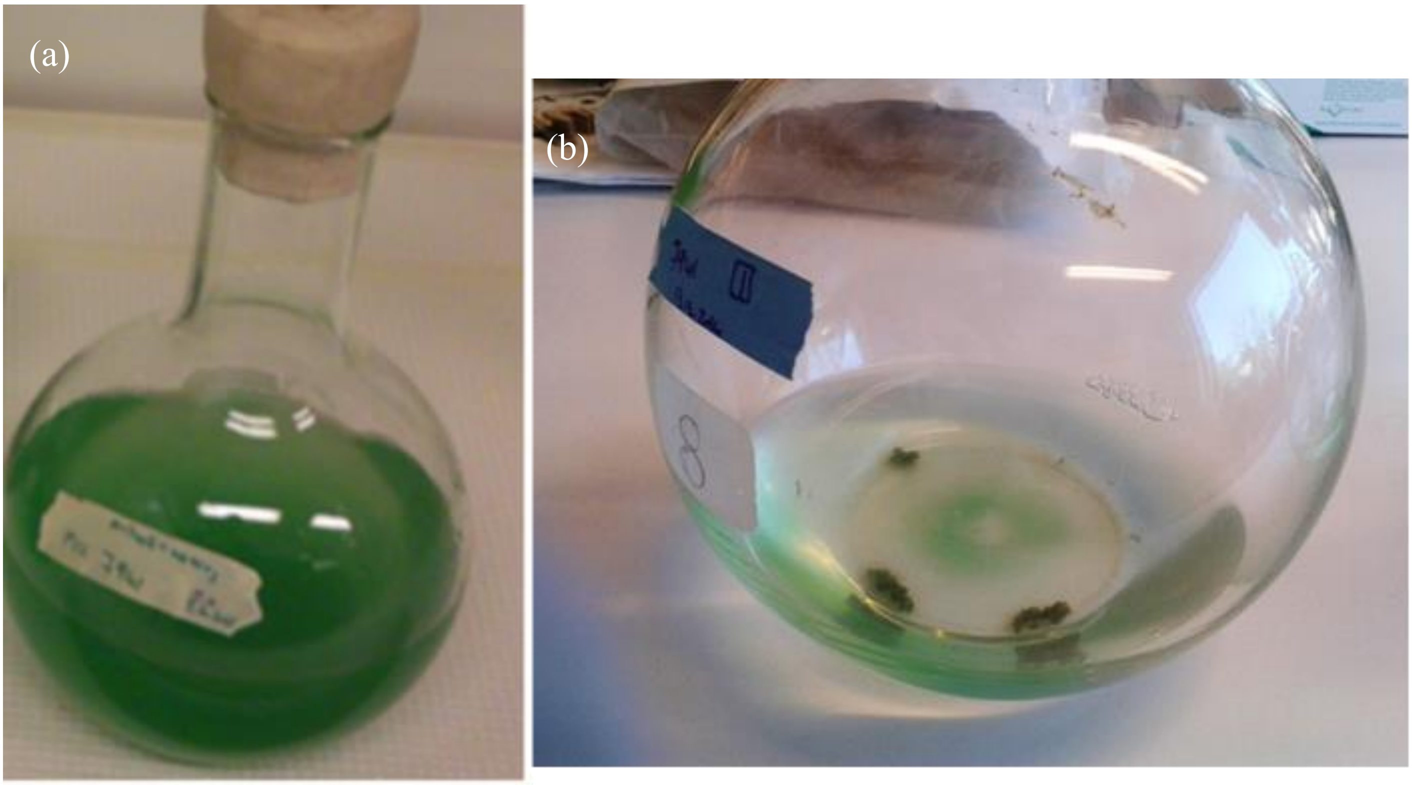
(a)
(b)

Supplementary figures legends:

Fig S1: Plaque formation in *Microcystis aeurignosa* PCC 7941 lawn.

Fig S2: Change in the *M. aeurignosa* PCC 7941 coculture appearance when exposed to heterotrophic strain. a: normal *Microcystis aeurignosa* PCC 7941 in liquid culture; b: *M. aeurignosa* PCC 7941 and *Stenotrophomonas rhizophila* EK20 coculture.
